# Related Effects of Methamphetamine on the Intestinal Barrier *via* Cytokines, and Potential Mechanisms by Which Methamphetamine May Occur on the Brain-Gut Axis

**DOI:** 10.3389/fmed.2022.783121

**Published:** 2022-05-10

**Authors:** Yuansen Li, Deshenyue Kong, Ke Bi, Huayou Luo

**Affiliations:** ^1^Department of Intestine and Hernia Surgery, The First Affiliated Hospital of Kunming Medical University, Kunming, China; ^2^NHC Key Laboratory of Drug Addiction Medicine, Kunming Medical University, Kunming, China; ^3^Yunnan Institute of Digestive Disease, The First Affiliated Hospital of Kunming Medical University, Kunming, China

**Keywords:** methamphetamine, intestinal barrier, tight junction (TJ), cytokines, the brain-gut axis

## Abstract

Methamphetamine (METH) is an illegal drug widely abused in many countries. Methamphetamine abuse is a major health and social problem all over the world. However, the effects of METH on the digestive system have rarely been reported. Previous studies and clinical cases have shown that METH use can lead to the impaired intestinal barrier function and severe digestive diseases. METH can cause multiple organ dysfunction, especially in the central nervous system (CNS). The gut microbiota are involved in the development of various CNS-related diseases *via* the gut-brain axis (GBA). Here, we describe the related effects of METH on the intestinal barrier *via* cytokines and the underlying mechanisms by which METH may occur in the brain-gut axis.

## Introduction

Methamphetamine (METH) abuse is a major public health and social problem throughout the world. METH is a widely abused illegal drug and is a highly addictive psychostimulant that can easily cross the blood–brain barrier (BBB) and cause severe brain damage (1), leading to neurological abnormalities and eventually mental disorders (2–4). Some of the symptoms of METH neurotoxicity include oxidative stress, excitatory toxicity, mitochondrial dysfunction, and microglial cell proliferation. METH can also cause acute, subacute, and chronic damage to the nervous system (5), the cardiovascular system, the respiratory system, the digestive system, and the teeth (6). At the same time, the adverse reactions of METH include hypertension, tachycardia, arrhythmia (7), psychosis (4), coronary artery and peripheral vascular spasm (7), insomnia (8), tremor, gastrointestinal disorder (9), and drug addiction.

Methamphetamine has different pharmacokinetic characteristics in different ways of use. Generally, it can enter the body by swallowing, injection, snorting, and smoking. METH is taken orally by the majority of people (10). The upper and lower digestive tracts are the first tissues to contact METH, so the research on the damage caused by METH to the digestive system is increasing day by day. It is one reason for writing this review.

According to previous studies and clinical cases, METH use can contribute to the formation and function of the digestive system. Vasoconstriction of the gastrointestinal tract is the most common physical change. It also includes abdominal pain or stomach pain, severe constipation or diarrhea, or alternating between constipation and diarrhea (9). In some cases, a significant reduction in gastrointestinal blood flow can contribute to serious complications, such as paralytic ileus, which if left untreated can lead to severe infections, gangrene, perforation of the intestinal wall, and water and electrolyte disturbances. In severe cases, intestinal infarction may develop into septic shock with multiple organ failures. Multiple studies have shown that damage to the intestinal mucosa and epithelial barrier resulting in increased intestinal permeability plays an important role in the pathophysiology of anxiety, stress, depression, cognitive decline, chronic fatigue, and eating and sleep disorders, all of which are clinically common among METH users (9). It can also be seen that the intestinal barrier plays an important role in the intestinal injury caused by METH. Recently, studies on METH and the intestinal barrier have been increasing and gaining attention. However, there is still no systematic treatment for intestinal barrier repair. Treatment of the intestinal barrier is currently limited to the disease itself.

## Intestinal Barrier

### Intestinal Barrier Composition

In this article, the intestinal barrier is defined as a dynamic, permeable physical and immune defense barrier composed of a variety of proteins and intestinal epithelial cells as well as epithelial cell secretions and a large number of immune cells below the intestinal epithelium. On the one hand, the intestinal barrier allows nutrients and certain fluids to be absorbed into the body to some extent; on the other hand, it also prevents toxins and bacteria from entering the body through the intestinal epithelium (11). The stability of the epithelial barrier function is a necessary condition to maintain the stability of the internal mucosal environment. In the subepithelial lamina propria, the balance between pro-inflammatory factors and anti-inflammatory factors plays a certain role in the maintenance of intestinal epithelial barrier function. The inflammatory microenvironment alters the structure and function of junctions between epithelial cells through both direct and indirect mechanisms, affecting epithelial barrier permeability.

**FIGURE 1:**
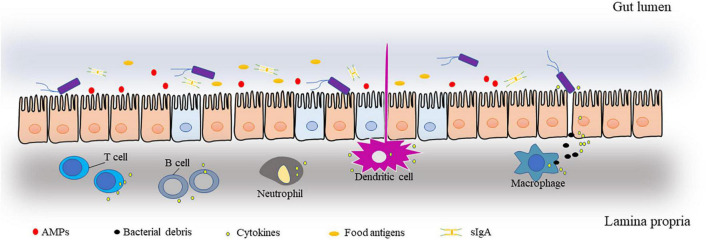
Schematic representation of the main components of the intestinal barrier.

Here, we divide the intestinal barrier into three interconnected and interrelated layers (Figure 1). (1) The external mucus layer is symbiotic with the intestinal flora and consists of antimicrobial proteins (AMPs) and secreted immunoglobulin A (sIgA), food antigens, mucinous proteins, digestive enzymes, etc. (2) A central single-celled layer with specialized epithelial cells is a single columnar epithelium that separates the body from the lumen environment. Epithelial cells are made up of several different cell types, such as Paneth cells and goblet cells, and these cells and their different functions form a tight barrier to protect the intestinal environment. The types of these cells and their roles in the intestinal barrier have been described in other reviews (12). (3) Lamina propria: home to innate and adaptive immune cells, such as T cells, B cells, macrophages, and dendritic cells. This layer is the body’s largest reservoir of immune cells and is responsible for clearing bacteria and viruses as well as dead cells, secreting inflammatory cytokines, and maintaining intestinal homeostasis.

Intestinal epithelial cells are connected by intercellular molecular connection complexes, such as tight junctions, adhesion junctions, desmosomes, and gap junctions. Molecular linkage complexes between intestinal epithelial cells play an important role in the entry of certain substances into the lamina propria or portal vein circulation.

In other reviews, the effects of various factors [such as dietary factors, alcohol, medication (NSAIDs and PPIs), smoking, and stress] on the intestinal barrier function (such as increased intestinal barrier permeability) (13), as well as methods and experiments for detecting intestinal barrier function are described in detail (13, 14).

### Tight Junction

The tight junction is a multi-protein composite [e.g., Occludin, Tricellulin, MarvelD3, Claudin protein family (15), JAM, ZO-1, ZO-2, ZO-3, cingulin, and symplekin (16, 17)] that connects the top end between epithelial cells and plays an important role in the intestinal barrier by allowing certain solutes and molecules to be transported but blocking the proteins, lipids, and microbial peptides (18, 19), strictly limiting the transportation of various harmful molecules (20). Therefore, any change in the tight junction structure may be harmful to the body. Disruption and/or degradation of TJ contribute to increased intestinal barrier permeability. Disorder of TJ protein expression/positioning and reduced intestinal barrier permeability lead to intestinal leakages and pathological state of the systemic diseases, such as inflammatory bowel disease (IBD), celiac disease, gastrointestinal tract infection, and chronic liver disease, autoimmune arthritis, multiple sclerosis, type 1 diabetes, and Parkinson’s disease. Therefore, barrier defects were significantly associated with abnormal systemic immune responses.

## Meth and Genomics of Gut

Transcriptome studies have been conducted to investigate changes in gene expression in intestinal tissues caused by METH. A total of 326 differentially expressed genes (DEGs) were identified in the transcriptome study of the meth-induced IBD mouse model (Table 1). Among them, 120 genes were upregulated and 206 genes were downregulated, suggesting that METH significantly affected the intestinal transcriptome of mice (21). DEGs obtained from the METH-induced IBD mouse model and bioinformatics analysis showed that METH may cause a specific type of IBD. These results provide new insights into the relationship between METH abuse and IBD. A new molecule or pathway may play a role when METH causes IBD, but whether it has practical significance for the clinical treatment of such patients and the treatment of IBD remains to be further studied.

**TABLE 1:** Bioinformatics analysis of differentially expressed genes in methamphetamine-induced inflammatory bowel disease mouse models.

The biological processes	Cellular catabolic processes, endocytosis, and autophagy
Molecular functions	Protein transferase, GTPase and proteinase activities, actin-binding, and protein-lipid complex binding
Pathway analysis	Bacterial invasion of epithelial cells, protein processing in the endoplasmic reticulum,
	Regulation of the actin cytoskeleton, and T-cell receptor signaling pathways
Main cellular components	The endoplasmic and endocytic vesicles, cytoskeleton,
	Adherens junctions, focal adhesions, cell body, and lysosomes

## Meth Contributes to Intestinal Barrier Damage Through Cytokines

Cytokine-mediated alterations in paracellular permeability in a variety of clinical and pathological conditions such as IBD, airway inflammation (22, 23), cystic fibrosis in asthma, and blood-brain barrier disorders (24). TNFα, IFN-γ, and interleukin play important roles in regulating tight junction integrity (25). Cytokine-mediated signaling interferes with tight junctions and increases intestinal barrier permeability, increasing tissue exposure to antigens in the gastrointestinal tract. When intestinal barrier function is disrupted, endotoxin (lipopolysaccharide LPS) is more likely to enter the circulatory system. In *in vitro* microglial cell studies, METH reduced the distribution and expression of toll-like receptor 4 (TLR4) and the production of pro-inflammatory factors after LPS action (26). This is different from the effects of alone METH on the TLR4 of microglial cell. In macrophages *in vitro* experiments [the phenotype or abundance of macrophages in the gut wall contribute to the development of the intestinal epithelium and the ability to sample gut antigens (27)], METH and LPS can significantly increase TNF-α, IL-1β, and IL-8 levels (signal transduction paths in NF-κ B, MAPK, and PI3-Akt are involved in mediated increased inflammatory responses) (28). The increase in cytokines or chemokines is usually greater than the increase in LPS treatment alone.

**FIGURE 2:**
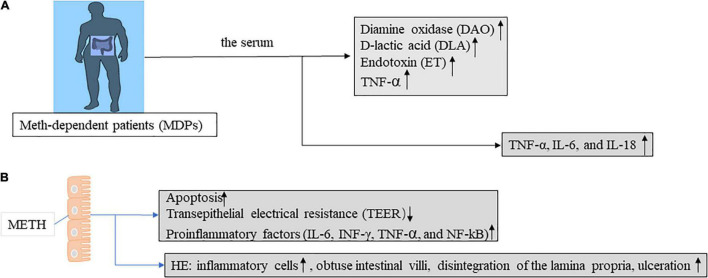
Intestinal barrier function was evaluated in mice and humans in the context of METH treatment. (A) Intestinal mucosal injury, intestinal wall permeability, and bacterial translocation are assessed by determining the serum levels of diamine oxidase (DAO), D-lactic acid (DLA), and endotoxin (ET) in Meth-dependent patients (MDPs). Abnormal levels of all these indicators reflect intestinal barrier dysfunction. (B) Intestinal epithelial cells of methamphetamine-treated mice are evaluated for intestinal barrier function by apoptosis, TEER, proinflammatory factors, and HE staining. Abnormal levels of all these indicators reflect intestinal barrier dysfunction.

Long-term methamphetamine use can cause changes in cytokine levels. Previous studies have reported that (29) serum levels of TNF-α, IL-6, and IL-18 were measured in 78 hospitalized long-term METH users. The TNF-α, IL-6, and IL-18 levels increased significantly (Figure 2A). Persistent inflammation in abusers further induces nerve damage, it is usually characterized by glial proliferation and increased cytokine levels (30). METH leads to BBB dysfunction directly or indirectly through the release of TNF-α and subsequent activation of NF-κB pathway (31). TNF-α not only disrupted the tight junctions between cells and led to an increase in intestinal barrier permeability, but also induced apoptosis of epithelial cells. Apoptosis accounts for approximately half (56%) of the TNF-α-induced increase in permeability, while the other half is caused by the degradation of the tight junctions (32). Many researchers believe that TNF-α is an important inflammatory mechanism leading to Crohn’s disease and other IBDs.

**FIGURE 3:**
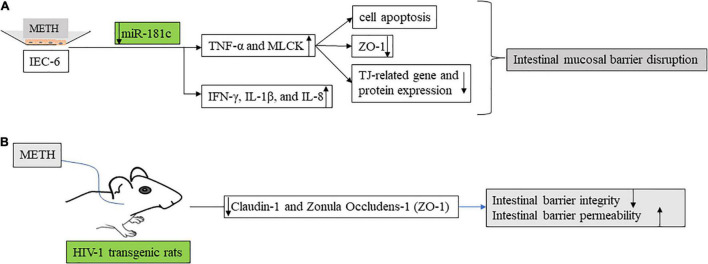
The effects of methamphetamine on intestinal barrier function. (A) In *in vitro* model, methamphetamine regulates Mir-181c. Anti-mir-181c promotes the secretion of TNF-α and MLCK, resulting in increased intestinal barrier permeability (decreased tight junction related protein) and epithelial cell apoptosis. IEC-6 is a well-established rat cell line with characteristics similar to those of intestinal epithelial cells and is extensively used as a surrogate for intestinal epithelial cells, for *in vitro* studies. MLCK, myosin light chain kinase. (B) Self-administered methamphetamine resulted in decreased colonic barrier function [Claudin-1 and Zonula occludens-1 (ZO-1)] and impaired colonic integrity in HIV-1 transgenic rats.

In animal models of METH, METH can cause significant intestinal barrier damage, increase pro-inflammatory factors (such as, IL-6, INF-γ, TNF-α, and NF-κB) (33), and induce intestinal tissue injury in mice (Figure 2B). The IFN-γ signaling pathway in intestinal glial cells is critical for intestinal homeostasis, and the IFNγ -EGC-CXCL10 axis plays a key role in immune response and tissue repair after infection. METH treatment increases IL-8 expression *in vitro* through the nuclear factor-κB pathway. Meanwhile, IL-8 activation of CXCR1 (C-X-C motif chemokine receptor 1) induces an increase in METH-related neuronal apoptosis *in vivo* and *in vitro* (34). Increased IL-8 was also observed in mouse intestinal epithelial cells cultured *in vitro* (33). In addition, cytokines (IFN-γ, IL-1β, and IL-8) in epithelial cells were increased in the METH *in vitro* model. In mouse models (Figure 3A), METH-induced spatial learning was impaired, and this effect was associated with reduced hippocampal IL-1β levels. Studies have shown that the loss of METH-related cognitive decline is linked to IL-1 (35). It provides a potential new therapeutic approach for the treatment of cognitive changes in individuals with METH abuse.

MicroRNAs have been shown to regulate epithelial barrier homeostasis by mediating the production of TNF-α and to participate in a variety of biological processes, such as cell proliferation, apoptosis, invasion, and metastasis, and stem cell differentiation, forming complex networks with important functions (36). In *in vivo* models, human diamine oxidase (DAO), D-lactic acid (DLA), and endotoxin (ET) levels are used to assess intestinal mechanical barrier function (Figure 2A). In an *in vitro* model, METH inhibits Mir-181c (miRNAs that regulate TNF-α) and then promotes TNF-α (TNF-α activates the MLCK promoter through the NF-κB pathway, and then promotes the increase of MLCK transcription, expression, and activity (37), and promotes the downregulation of ZO-1 protein expression and changes in junction location) (38) secretion, thereby increasing intestinal barrier permeability and epithelial cell apoptosis (33) (Figure 3A). This provides a new direction for the treatment of intestinal damage caused by METH, and microRNA regulation has a greater application prospect in future therapeutic strategies to prevent intestinal barrier dysfunction compared with available targeted protein drugs.

**FIGURE 4:**
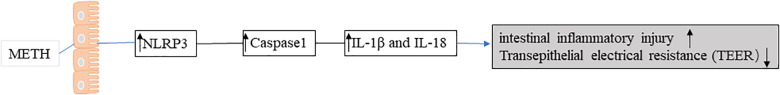
Methamphetamine (METH) promotes the overexpression of NLRP3 inflammasome and induces intestinal inflammatory injury.

Previous studies in a model of chronic METH abuse in rhesus monkeys have shown that METH can cause damage to the intestinal mucosal barrier, resulting in a high risk of intestinal infection. In addition, METH enhanced the expression of chemokine and chemokine receptor-related genes and promoted the apoptosis of tissue cells and the pro-inflammatory pathway (39). Recent studies have shown that METH promotes the overexpression of NLRP3 inflammasome and induces intestinal inflammatory injury (40) (Figure 4), NLRP3 is widely expressed in the gastrointestinal tract and can be found in epithelial cells at mucosal sites, which are important for maintaining environmental balance in the gut (41). Blocking NLRP3 inflammasome activation partially prevents inflammatory damage.

When it comes to METH abuse, it is necessary to mention the special group of people infected with HIV. Amanda L. Persons reported (42) reduced colonic barrier function and impaired colonic integrity in HIV-1 transgenic rats as a result of self-medicated METH (Figure 3B). METH abuse is a common situation among HIV-infected people. HIV infection itself causes certain damage to the intestine. In the self-drug model of HIV transgenic rats, the intestinal barrier permeability and endotoxin level of the colon of rats increased after METH addiction (42). At the same time, METH use and sexual behavior contribute to chronic HIV-1 infection and intestinal disorders in young men who have sex with men (43, 44). METH abuse in men infected with HIV is associated with a microbiological imbalance of pro-inflammatory bacteria, including some with neuroactive bacteria and bacteria thought to be associated with adverse HIV outcomes (44).

Numerous studies have shown that multiple pro-inflammatory factors increase in METH users, suggesting that METH-aggravated inflammation may be a common feature of infectious disease in METH abusers. Inflammatory factors have also been shown to disrupt intestinal barrier function in a variety of ways (tight junctions or apoptosis of intestinal epithelial cells). Anti-inflammatory factor drugs may play a role in protecting the intestinal barrier. The contribution of METH to inflammatory factors is not only limited to intestinal barrier injury but also affects diseases related to intestinal barrier function, although it has not been further confirmed. However, understanding the molecular and barrier function roles of disrupting and stabilizing intestinal epithelial TJs and understanding the development of inflammation may help to treat patients with intestinal barrier disorders associated with inflammation, or to prevent the development of inflammation. Therefore, bowel barrier therapy has good prospects in terms of improving a variety of diseases and cytokine regulation.

## The Effects of Meth on the Gut-Brain Axis

Methamphetamine can enhance the action of monoamines because METH has a similar chemical structure to monoamines and can directly bind to and activate the receptors of these compounds (45). Different doses of METH promote the release or enhancement of monoamines in different ways. In addition, METH attenuated monoamine metabolism. There is evidence that METH blocks the reuptake of catecholamines leading to an increase in the effects of norepinephrine. Most of the peripheral effects of norepinephrine released by METH are caused by the rapid and sustained release of catecholamines and stimulation of α and β adrenergic receptors (α 1 receptors cause peripheral vascular contraction, leading to hypertension and visceral ischemia). METH is known to have a sympathetic-like effect, leading to constriction of blood vessels in the visceral circulation. The rapid and sustained release of norepinephrine after METH use causes arterial constriction. Effects are also seen in the intestinal mesenteric blood vessels, leading to acute intestinal ischemia.

**FIGURE 5:**
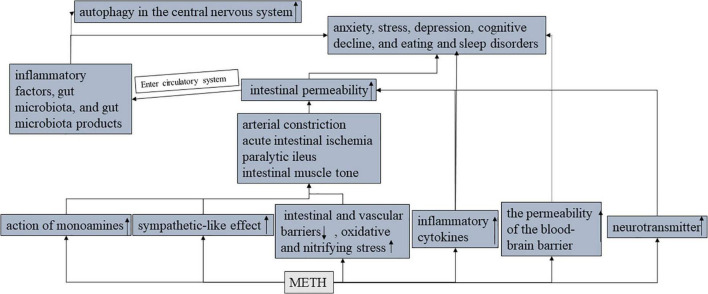
The effects of METH on the gut-brain axis.

The most common harmful effects of METH are seen in neurological and circulatory damage or disease. However, one case of paralytic ileus caused by METH was diagnosed (46). The mechanism may be that METH promotes the release of norepinephrine and dopamine, and the activation of the dopamine-1 receptor leads to a significant decrease in intestinal contractility and motor capacity. In addition, norepinephrine alters the intestinal nervous system, leading to a decrease in intestinal muscle tone. Intestinal obstruction is associated with intestinal and vascular barriers (47), oxidative stress, and nitrifying stress. METH-mediated neurotransmitter release also promotes the production of oxidative stress molecules [reactive nitrogen species (ROS) and reactive nitrogen species (RNS)], which can lead to the injury and death of intestinal neurons and intestinal barrier dysfunction. Intestinal mucosal barrier dysfunction increases intestinal permeability and plays an important role in the pathophysiology of anxiety (48), stress (49), depression (50), cognitive decline (51), and eating and sleep disorders (52) (Figure 5).

Disruption of the intestinal barrier (damage of intestinal epithelial cells and disruption of tight junctions) results in the leakage of inflammatory factors, gut microbiota, and gut microbiota products from the lumen into the circulatory and lymphatic systems (53). Gut microbes, as well as their metabolites, are produced in the vicinity of intestinal epithelial cells and therefore have a significant impact on intestinal barrier function and immune response (12). The human gastrointestinal tract contains countless types of microbes, and the relationship between the microbes and the host is very close. At the same time, METH use increases the permeability of the BBB (31), and these intestinal microbial components have the ability to enter the brain (3). Gut bacteria collaborate with their animal hosts to regulate immune, metabolic, and nervous system development and function through dynamic two-way communication along the “gut-brain axis (54).” Besides, the intestinal flora may play an important role in the communication between the gut and the brain, for example, the gut microbiome plays an important role in anxiety and depression (55, 56). In animal models of phenethylamine-induced hyperthermia (PIH), certain changes in gut bacteria promote the bidirectional regulation between the brain and gut (57) (PIH). In METH treated mouse models, METH increased the relative abundance of pathogenic bacteria (58), promoted intestinal inflammation, and reduced intestinal TJ protein expression, but decreased the relative abundance of probiotics and changed fecal metabolites. METH exposure enhances the intestinal autophagy flora, promotes the accumulation of fecal metabolites in the autophagy pathway, and induces autophagy in the central nervous system (59).

Methamphetamine abusers have increased intestinal inflammatory biomarkers, including inflammatory cytokines (already mentioned above), which have been shown to enter the brain and interact with areas of pathophysiology related to depression (60), such as neurotransmitter metabolism, neuroendocrine function, and neuroplasticity.

## Summary and Future Directions

There has been little progress in the treatment of common gastrointestinal symptoms, such as gastrointestinal pain, diarrhea, and bloating. As a result, there is a growing interest in gut barrier research. The role of intestinal barrier function is believed to be important, but there are still many unresolved questions because there is no clear gold standard for barrier function testing and the clinical significance and relevance of the existing multiple methods for measuring intestinal barrier function is unclear.

The effect of METH on the intestinal barrier does exist (the exact mechanism is still unclear). METH use has been shown to cause changes in intestinal barrier function and permeability (such as intestinal epithelial cell apoptosis and intercellular TJ damage), as well as cause some abdominal complications. The digestive system can be thought of as the body’s second “brain.” A number of cumulative observations suggest that a number of neurological disorders, such as METH-related changes, may be associated with increased intestinal barrier permeability, and that these relatively minor disturbances can be reversed by counteracting the associated inflammatory pathways and protecting the intestinal barrier. It is important to study the characteristics of the intestinal barrier and the pathways between intestinal epithelial cells to solve the problems related to the brain-gut axis, which provides a basis for the detection of parenteral diseases. In addition, part of the gut-brain axis includes the effects of gut flora on the brain, behavior, and health. For example, taking probiotics can treat certain aspects of depressive and anxious behavior (61–65). Some probiotics may improve intestinal barrier dysfunction, but it is unclear whether intestinal barrier therapy is associated with anxiety and depression. Thus, gut microbiota and/or metabolites may affect the health and neurological function of the host. Moreover, gastrointestinal dysfunction is more common in the early stages of Parkinson’s disease than other systemic symptoms. In Hye-young Sung’s questionnaire, 88.9% of 54 selected patients reported intestinal symptoms before the early motor symptoms of Parkinson’s disease (66). This suggests that intestinal alterations may help in the early diagnosis of neurological diseases.

Increased intestinal epithelial permeability is due to increased paracellular transport, apoptosis, or transcellular permeability. Any inflammatory process can compromise barrier integrity; for example, anti-TNF-α therapy reduces mucosal inflammation and restores intestinal permeability in patients with IBD. Cells at the gut barrier are exposed to microbial and METH factors that may promote the production of inflammatory factors either by directly damaging cells or by overactivating inflammatory processes. Cytokines and chemokines are neuroimmune factors expressed in neurons, astrocytes, and microglia, which show promising application prospects in clinical neuroinflammation, neuronal injury, and behavioral disorders. Inflammatory factors may serve as potential therapeutic targets for METH use disorders in the future.

Impaired intestinal barrier function is associated with many intestinal and systemic disease states. Unfortunately, most current clinical data are correlated, making it difficult to distinguish cause from effect when interpreting the significance of barrier loss. The effect of restoring barrier function on improving clinical manifestations of local gastrointestinal disease or systemic disease has also not been demonstrated. There is currently no treatment that targets the epithelial barrier. To achieve this goal, the regulatory mechanisms of barriers should be better understood. Clinicians should be aware of the possibility of barrier dysfunction in gastrointestinal diseases and as a target for future treatment.

## Author Contributions

YL, KB, HL, and DK conceived and designed the manuscript and prepared the manuscript. YL, HL, DK, and KB performed the literature search.YL and DK did the picture making. KB, YL, DK, and HL revised the manuscript. All authors read and approve the final version of the manuscript.

## Conflict of Interest

The authors declare that the research was conducted in the absence of any commercial or financial relationships that could be construed as a potential conflict of interest.

## Publisher’s Note

All claims expressed in this article are solely those of the authors and do not necessarily represent those of their affiliated organizations, or those of the publisher, the editors and the reviewers. Any product that may be evaluated in this article, or claim that may be made by its manufacturer, is not guaranteed or endorsed by the publisher.
